# Features of Lipid Disorders in Cardiovascular–Kidney–Metabolic Syndrome

**DOI:** 10.3390/ijms27188317

**Published:** 2026-09-18

**Authors:** Alexandr Ceasovschih, Yusuf Ziya Şener, Mustafa Arici, Mariia Cherska, Vanessa Bianconi, Malik Ejubović, Stanislav Kotlyarov, Vladimir Ristovski, Ahmet Yetkin, Pelin Golforoush, Tatyana Storozhenko, Fotios Barkas, Alexandru Corlateanu, Pradeesh Sivapalan, Tea Gamezardashvili, Antonio M. Gotto, Laurentiu Sorodoc, Victorita Sorodoc

**Affiliations:** 1 Grigore T. Popa University of Medicine and Pharmacy, 700115 Iasi, Romania; 2 Department of Cardiology, Thoraxcentrum, Erasmus MC, 3015 CE Rotterdam, The Netherlands; 3 Department of Internal Medicine, Yavuzeli District State Hospital, 27907 Gaziantep, Türkiye; 4 Department of Nephrology, Hacettepe University, 06800 Ankara, Türkiye; 5 State Institution “V.P. Komisarenko Institute of Endocrinology and Metabolism of the National Academy of Medical Sciences of Ukraine”, 04114 Kyiv, Ukraine; 6 Department of Medicine and Surgery, University of Perugia, 06132 Perugia, Italy; 7 Department of Internal Medicine, Cantonal Hospital Zenica, 72000 Zenica, Bosnia and Herzegovina; 8 University of Zenica, 72000 Zenica, Bosnia and Herzegovina; 9 Department of Nursing, Ryazan State Medical University, 390026 Ryazan, Russia; 10 Department for Invasive and Interventional Cardiology, City General Hospital “8th of September”, 1000 Skopje, North Macedonia; 11 Department of Cardiology, Suşehri District State Hospital, 58600 Sivas, Türkiye; 12 The Francis Crick Institute, 1 Midland Rd, London NW1 1AT, UK; 13 Cardiovascular Center Aalst, 9300 Aalst, Belgium; 14 Department of Prevention and Treatment of Emergency Conditions, L.T. Malaya Therapy National Institute NAMSU, 61039 Kharkiv, Ukraine; 15 Department of Internal Medicine, Faculty of Medicine, School of Health Sciences, University of Ioannina, 45110 Ioannina, Greece; 16 Department of Respiratory Medicine and Allergology, State University of Medicine and Pharmacy “Nicolae Testemitanu”, 2004 Chisinau, Moldova; 17 Department of Clinical Medicine, Faculty of Health and Medical Sciences, University of Copenhagen, DK-2200 Copenhagen, Denmark; 18 Copenhagen Respiratory Research, Department of Medicine, Copenhagen University Hospital—Herlev and Gentofte, DK-2900 Copenhagen, Denmark; 19 Remedical Hospital, 9/16’ Tsinandali Str., 1044 Tbilisi, Georgia; 20 Georgian Atherosclerosis Association, 9’ L. Managadze Str., 0144 Tbilisi, Georgia; 21 Weill Medical College, Cornell University, New York, NY 10065, USA

**Keywords:** cardiovascular–kidney–metabolic, dyslipidaemia, lipid disorders, lipid-lowering therapy

## Abstract

Cardiovascular–kidney–metabolic (CKM) syndrome is driven by the interplay of visceral adiposity, insulin resistance, chronic inflammation, oxidative stress, and progressive kidney dysfunction. These mechanisms generate a distinct atherogenic dyslipidaemic phenotype characterised by elevated triglycerides (TG), reduced high-density lipoprotein-cholesterol (HDL-C), increased apolipoprotein B (apoB)-containing lipoproteins, remnant cholesterol (RC) accumulation, small dense low-density lipoprotein (LDL) particles, and frequently elevated lipoprotein(a) [Lp(a)]. Such lipid abnormalities contribute to accelerated atherosclerosis and early renal deterioration. In turn, kidney dysfunction further amplifies lipid abnormalities. These pathways underscore the need for a timely and comprehensive lipid assessment using apoB, non-HDL-C, RC, and Lp(a) within the CKM framework. Effective lipid management is crucial for CKM syndrome treatment, with the potential to reduce residual cardiovascular and renal risk and improve long-term clinical outcomes. In this narrative review, we examine current evidence on the pathophysiological mechanisms underlying CKM-related dyslipidaemia and provide a practical framework for its assessment and management. Management requires a multimodal approach tailored to the patient’s lipid phenotype and overall cardiovascular risk, incorporating a range of lipid-lowering strategies including statins, ezetimibe, PCSK9 inhibitors, bempedoic acid, fibrates, icosapent ethyl, and emerging therapies targeting apoC-III, ANGPTL3, and Lp(a). Beyond lipid lowering, lifestyle interventions and cardiometabolic therapies such as glucagon-like peptide-1 receptor agonists, sodium-glucose cotransporter 2 inhibitors, and finerenone may provide complementary cardiometabolic and renal protection.

## 1. Introduction

The American Heart Association defines cardiovascular–kidney–metabolic (CKM) syndrome as a systemic disorder characterised by pathological interactions among metabolic risk factors, chronic kidney disease (CKD), and the cardiovascular system, resulting in multiorgan dysfunction and increased rates of overall morbidity and mortality. A key feature of CKM syndrome is dysfunctional adipose tissue, particularly excess visceral adiposity, which releases proinflammatory and prooxidative mediators that contribute to cardiovascular and renal injury [1,2,3].

Atherogenic dyslipidaemia is a hallmark of CKM syndrome and is characterised by elevated triglycerides (TG), reduced high-density lipoprotein cholesterol (HDL-C), increased concentrations of apolipoprotein B (apoB)-containing lipoproteins, and a predominance of small dense low-density lipoprotein (LDL) particles [4,5]. This atherogenic lipid profile plays a central role in the development and progression of atherosclerosis and is associated with adverse cardiovascular and renal outcomes in patients with CKM syndrome [1,2,3].

Lipid abnormalities in CKM syndrome arise from a complex interplay of pathophysiological processes. Insulin resistance disrupts lipid metabolism and promotes an atherogenic lipid profile characterised by elevated levels of TGs and small dense LDL particles and reduced HDL-C. Dysfunctional adipose tissue releases pro-inflammatory cytokines and adipokines, exacerbating lipid dysregulation and promoting persistent, low-grade systemic inflammation. Metabolic dysfunction-associated steatotic liver disease (MASLD) worsens lipid metabolism by increasing systemic inflammation and insulin resistance. Increased oxidative stress promotes endothelial dysfunction, vascular inflammation, and atherosclerosis, contributing to poor cardiovascular and renal outcomes in CKM syndrome [1,6].

Lipid abnormalities, in addition to atherosclerotic cardiovascular disease, contribute to heart failure with preserved ejection fraction (HFpEF) and atrial fibrillation via inflammatory and fibrotic mechanisms. The atherogenic lipid profile can emerge early in the course of CKD, underscoring the necessity for prompt identification and care [7,8].

This review aims to (i) characterise the specific lipid phenotypes associated with CKM syndrome, (ii) summarise the pathophysiological mechanisms and clinical implications, and (iii) provide an overview of current and emerging therapeutic strategies, including both lipid-lowering and broader cardiometabolic interventions, within the CKM framework. Understanding the mechanisms underlying lipid abnormalities and the evolving therapeutic landscape is therefore essential to reducing residual cardiovascular and renal risk and improving long-term clinical outcomes in the CKM population.

Although this review is narrative rather than systematic, a structured search strategy was employed to ensure comprehensive coverage of the available evidence and to enhance the transparency in the selection process. Literature searches were conducted using PubMed/MEDLINE, Scopus and Google Scholar. The search was conducted using combinations of the following keywords: “cardiovascular–kidney–metabolic (CKM) syndrome”, “dyslipidaemia”, “chronic kidney disease”, “cholesterol”, “lipid-lowering therapy”, “triglycerides”, “lipoproteins”, and “insulin resistance”, among others. Relevant publications included original research studies, narrative and systematic reviews, and clinical case reports. Priority was given to studies published within the last 10 years, while earlier studies were included where necessary to provide historical context and foundational clinical and preclinical research.

Existing reviews have largely focused on the general principles of CKM syndrome, with less emphasis on the specific pathophysiological mechanisms of dyslipidaemia and their clinical implications across the CKM continuum. This review provides an in-depth analysis of these mechanisms, including the role of insulin resistance, MASLD, inflammation, oxidative stress, renal dysfunction, lipotoxicity, ectopic fat, and uremic dyslipidaemia, alongside a discussion of new and emerging lipid-modifying therapies in the context of CKM syndrome.

## 2. Pathophysiology of Lipid Disorders in CKM Syndrome

### 2.1. Atherogenic Dyslipidaemia

Dyslipidaemia is a key component of CKM syndrome. Traditional atherogenic dyslipidaemia in patients with CKD takes on additional characteristics due to impaired renal function and uremic toxins [9].

One of the most common quantitative abnormalities in lipid metabolism in patients with CKD is elevated TG levels [10]. Under normal physiological conditions, TG-rich very low-density lipoproteins (VLDL) undergo lipolysis by lipoprotein lipase (LPL), generating intermediate-density lipoproteins (IDL). In CKD, LPL activity is reduced, while levels of apolipoprotein C-III (apoC-III), a competitive inhibitor of LPL-mediated lipolysis, are increased [11,12,13]. Impaired metabolism of apoC-III in plasma is a characteristic feature of dyslipidaemia in moderate CKD. Excessive accumulation of apoC-III results from impaired catabolism and is associated with an increased risk of cardiovascular disease [11,14].

CKD is also associated with an increased proportion of small dense LDL particles, which are highly atherogenic [15,16,17]. Notably, in the context of CKM syndrome, elevated levels of small, dense LDL particles are associated not only with cardiovascular events but also with an increased risk of multimorbidity, defined as the presence of two or more cardiorenal and metabolic conditions, independent of total LDL-C levels [18].

Another important feature of dyslipidaemia in CKD is elevated lipoprotein(a) [Lp(a)], likely due to impaired renal catabolism of this lipoprotein [19,20,21]. Lp(a) levels may increase early in the course of declining renal function, even during the initial stages of reduced glomerular filtration rate [21,22]. Elevated Lp(a) concentrations in patients with CKD are significantly associated with an increased risk of cardiovascular disease [23].

Furthermore, dyslipidaemia in patients with CKD is characterised by the accumulation of remnant cholesterol (RC) [24]. Due to their small size, RC particles can easily penetrate the arterial wall and be engulfed by macrophages, leading to the formation of foam cells [25]. Elevated levels of RC are an independent risk factor for the progression of CKM syndrome and increased cardiovascular risk [26]. The ratio of RC to C-reactive protein (CRP), known as the RC inflammatory index (RCII), is considered an epidemiological biomarker for the risk of cardiovascular disease and mortality [27,28].

When assessing atherogenic risk, the number of atherogenic particles may be more informative than their cholesterol content. Each particle of VLDL, IDL, LDL, remnants, and Lp(a) contains one molecule of apoB; therefore, plasma apoB concentration provides a direct measure of the total number of circulating atherogenic particles [29,30]. In contrast, LDL-C reflects the cholesterol mass within LDL particles rather than their number, while non-HDL-C captures the cholesterol content of all apoB-containing lipoproteins. Non-HDL-C can be readily assessed in the non-fasting state and is calculated as total cholesterol minus HDL-C, without requiring a triglyceride-dependent estimation of LDL-C. Like LDL-C, however, non-HDL-C reflects the cholesterol content of circulating atherogenic lipoproteins rather than directly measuring their particle number [29,31]. Notably, the cholesterol content of individual lipoprotein particles is variable: small, dense particles are relatively cholesterol-depleted, while larger particles contain more cholesterol. Consequently, the same value of LDL-C or non-HDL-C may correspond to different numbers of circulating atherogenic particles. This discordance is clinically relevant, because cardiovascular risk may be more closely related to the number of atherogenic particles reflected by apoB than to their cholesterol content. Therefore, apoB may be a more accurate marker of atherogenic risk than LDL-C and non-HDL-C in the context of CKM syndrome [32,33]. Consistent with current recommendations, apoB should be interpreted as a complementary measure alongside LDL-C and non-HDL-C for detecting residual particle-related risk after standard lipid targets have been achieved, particularly in patients with diabetes, elevated triglycerides, or very low achieved LDL-C levels, rather than as a universal replacement for standard lipid targets [30]. The characteristic lipid profile of CKM syndrome, including hypertriglyceridaemia, accumulation of TG-rich lipoproteins, and a predominance of small, dense LDL particles, further increases the likelihood of discordance between cholesterol-based measurements and apoB. Consequently, LDL-C and, to a lesser extent, non-HDL-C, may underestimate the total number of atherogenic particles [29,31]. In a large population-based study, individuals with discordantly low LDL-C and high apoB levels had the most unfavourable metabolic phenotype and the poorest renal function [34]. Therefore, apoB and non-HDL-C more accurately reflect plasma atherogenic burden and residual cardiovascular risk compared to LDL-C alone in patients with CKM syndrome [30,31,33].

HDL-C levels are reduced in CKD due to several mechanisms, including impaired cholesterol reverse transport, reduced concentrations of lecithin-cholesterol acyltransferase [35], and increased cholesteryl ester transfer protein activity [36]. In addition, late-stage CKD, characterised by systemic oxidative stress and inflammation, is associated with alterations in HDL composition and function, resulting in reduced protective activity and the emergence of dysfunctional or potentially proatherogenic HDL particles [37,38,39,40]. Common compositional changes in HDL in CKD include elevated levels of serum amyloid A and apoC-III, as well as a significant decrease in the activity of HDL-associated paraoxonase 1 [38,41].

The heterogeneity of lipid disorders observed within the CKM spectrum is also significantly influenced by genetic factors. The highly polygenic nature of dyslipidaemia has been underscored by the identification of numerous loci associated with circulating LDL-C, HDL-C, triglycerides, and other atherogenic lipoprotein traits in genome-wide association studies (GWAS). Genes that are involved in critical pathways of lipid metabolism, such as LDLR, APOB, PCSK9, APOE, LPL, ANGPTL3/4, CETP, and SORT1, have been implicated in extensive multi-ancestry analyses. It is crucial to note that genetic studies have not only enhanced comprehension of lipid biology but have also facilitated the identification and validation of therapeutic targets. PCSK9 and ANGPTL3 are two prominent examples. More recent GWAS that incorporate comprehensive lipid and metabolic phenotypes further elucidate the genetic connections between triglyceride-rich lipoproteins, insulin resistance, and cardiometabolic disease. However, the transferability of genetic risk estimates across ancestral populations remains a critical factor, which underscores the necessity of a greater diversity in GWAS and the cautious application of polygenic risk scores in clinical risk stratification [42].

### 2.2. Insulin Resistance and MASLD

Insulin resistance has a central pathogenic role in CKM syndrome by connecting lipid metabolism disorders with renal dysfunction and hepatic steatosis [43,44,45,46]. Indeed, insulin resistance disrupts podocyte integrity, enhances tubular gluconeogenesis, and disrupts sodium balance, leading to albuminuria, hypertension, and hyperglycaemia. In addition, renal lipotoxicity associated with insulin resistance and hyperinsulinaemia leads to podocyte dysfunction, mesangial expansion, and tubular damage, exacerbating nephropathy within the context of CKM syndrome [47]. Furthermore, insulin resistance contributes to hepatic metabolic dysfunction by promoting lipid accumulation in hepatocytes and triggering a cascade of inflammatory and fibrotic reactions leading to hepatic steatosis and its progression. Hepatic steatosis, in turn, promotes the secretion of atherogenic lipoproteins and pro-inflammatory cytokines [48]. Under physiological conditions, the liver takes up circulating free fatty acids from the peripheral bloodstream and converts them into TGs. However, chronic lipid overload promotes the secretion of TGs in the form of atherogenic VLDL [49,50,51,52].

The heterogeneity of MASLD also has important therapeutic implications, with emerging treatments targeting specific aspects of hepatic metabolic dysfunction. Resmetirom, a selective thyroid hormone receptor-β agonist, is the first licenced pharmacological treatment for non-cirrhotic MASH with moderate to advanced fibrosis (F2–F3). By targeting hepatic thyroid hormone signalling, resmetirom enhances lipid metabolism and has been shown to promote steatohepatitis resolution and improve fibrosis, highlighting the potential of targeting hepatic metabolic dysfunction as part of the broader therapeutic approach to CKM syndrome [53].

Thus, insulin resistance and the associated hyperinsulinemia create a vicious cycle in which renal injury and hepatic steatosis can further exacerbate one another, accelerating the progression of CKM syndrome.

### 2.3. Inflammation and Oxidative Stress

Chronic systemic inflammation represents another important link between the components of CKM. Macrophage infiltration of visceral adipose tissue promotes the secretion of pro-inflammatory cytokines (e.g., TNF-α, IL-6), which further promote insulin resistance in the liver, muscle, and adipose tissue [54,55]. These inflammatory signals can activate NF-κB in the vascular wall and kidneys, triggering a cascade of reactions leading to endothelial dysfunction and fibrosis. Furthermore, visceral adipose tissue is considered an active endocrine organ, secreting a wide range of signalling molecules known as adipokines. An imbalance between pro- and anti-inflammatory adipokines plays a key role in systemic inflammation, insulin resistance, and target organ injury [56,57].

The gut microbiota has also emerged as a potential contributor to CKM syndrome and an essential regulator of metabolic and cardiovascular health. While microbiota-derived metabolites can directly influence host lipid and glucose metabolism, gut dysbiosis may compromise intestinal barrier integrity and promote systemic inflammation by increasing the translocation of microbial products. In particular, trimethylamine N-oxide (TMAO), generated from dietary precursors through gut microbial and hepatic metabolism, has been associated with atherosclerosis, cardiovascular events and impaired kidney function. In contrast, beneficial microbial metabolites, including short-chain fatty acids, contribute to intestinal barrier integrity, immune homeostasis, and metabolic regulation. CKD may further disrupt the gut–kidney–cardiovascular axis by altering gut microbial composition and promoting the accumulation of microbiota-derived uremic toxins, including indoxyl sulphate and p-cresyl sulphate. These observations indicate that alterations in the microbiome may represent an additional mechanistic link between dyslipidaemia, inflammation, metabolic dysfunction, and cardiorenal disease across the CKM continuum [58,59].

Oxidative stress caused by hyperglycaemia and dyslipidaemia leads to the formation of oxidised lipoproteins (oxLDL). OxLDL not only accelerates atherogenesis but may also directly contribute to glomerular injury [60]. Renal dysfunction can further exacerbate systemic inflammation and oxidative stress, reinforcing this pathological cycle and accelerating the progression of CKM [61]. Clinical data indicate that the CRP–triglyceride–glucose index (CTI), which integrates metabolic and inflammatory signals, is associated with an increased risk of CVD in patients with CKM syndrome [62].

### 2.4. Effects of Kidney Dysfunction on Dyslipidaemia

As noted above, dyslipidaemia in CKD encompasses a complex range of lipid abnormalities. These changes become more pronounced as kidney dysfunction progresses and are further modified by conditions such as nephrotic syndrome or dialysis therapy.

Uremia promotes the carbamylation and oxidation of lipoproteins, generating highly atherogenic particles that are less readily recognised by LDL receptors and are actively taken up by macrophages via scavenger receptors [63,64,65]. Carbamylation occurs when reactive isocyanate, formed spontaneously from urea accumulating in the blood of patients with CKD, binds to plasma proteins. This process is considered one of the potential pathophysiological mechanisms linking CKD with increased risk of cardiovascular disease [65]. This form of “uremic dyslipidaemia” therefore differs qualitatively from “metabolic dyslipidaemia”.

There is also growing interest in the role of bioactive lipids, particularly ceramides, in the pathogenesis of CKM syndrome [60]. Ceramides are sphingolipids involved in the regulation of inflammation, apoptosis, insulin resistance, and cellular stress [66]. Elevated levels of certain types of ceramides are associated with a high risk of cardiovascular disease, diabetes, and CKD [67,68]. Plasma ceramide levels are associated with CKD independently of coronary artery disease, diabetes, and other established cardiovascular risk factors [69]. Ceramides partially mediate the association between CKD and coronary microvascular dysfunction [70]. Hyperlipidaemia and elevated ceramide levels frequently co-exist, and saturated fatty acids stimulate ceramide accumulation by enhancing de novo ceramide synthesis [67,71]. Ceramides have multiple potentially deleterious effects on renal and metabolic function. Elevated ceramide levels are associated with renal inflammation in CKD and can activate pro-fibrotic pathways, including TGF-β signalling, leading to extracellular matrix accumulation and fibrosis [72,73]. Ceramides affect the activity of the electron transport chain, impair mitochondrial function, and further contribute to renal cellular injury and CKD progression [67,74]. Furthermore, ceramides exacerbate systemic insulin resistance and inflammation [75,76], providing an additional link between the metabolic, cardiovascular, and renal components of CKM syndrome.

Thus, lipid abnormalities in CKM syndrome represent a complex, multifactorial phenotype arising from the interplay between metabolic dysfunction, inflammation, oxidative stress, hepatic steatosis, and kidney dysfunction. These changes contribute to residual cardiovascular risk even when LDL-C levels are within target ranges, helping explain why standard lipid-lowering therapy with statins does not fully eliminate atherosclerotic risk in patients with CKM syndrome.

### 2.5. Lipotoxicity and Ectopic Fat Deposition

Lipotoxicity and ectopic fat deposition are considered important pathogenic mechanisms of CKM syndrome. In addition to subcutaneous adipose tissue, which serves as a lipid storage site during a positive energy balance, excess lipid can accumulate in organs not primarily specialised for lipid storage, including the liver, pancreas, kidneys, heart, and blood vessels. This process, known as ectopic fat accumulation, is increasingly recognised as an important feature of metabolic dysfunction [77,78].

Ectopic fat accumulation in the myocardium and pericardial adipose tissue plays a key role in the development of cardiovascular complications [60]. At the molecular level, the key mechanisms of lipotoxicity are the induction of endoplasmic reticulum stress and mitochondrial dysfunction. Epicardial adipose tissue is metabolically active and capable of secreting pro-inflammatory cytokines and adipokines. This contributes to endothelial dysfunction, coronary inflammation, and the progression of atherosclerosis [79,80,81].

The kidneys are also an important target for lipotoxicity [82]. Ectopic lipid accumulation in the kidneys, historically described as “fatty kidney,” is now increasingly recognised as a feature of fatty kidney disease [83]. In CKM syndrome, excess free fatty acids can induce mitochondrial dysfunction, the formation of reactive oxygen species, endoplasmic reticulum stress, and the activation of pro-inflammatory pathways in the kidney [82]. Lipids contributing to renal ectopic accumulation include free fatty acids, TGs, and cholesterol [84,85]. Renal lipotoxicity can impair podocyte and proximal tubular epithelial cell function and promote tubulo-interstitial damage, contributing to glomerular and tubular injury [84,86].

**Figure 1:**
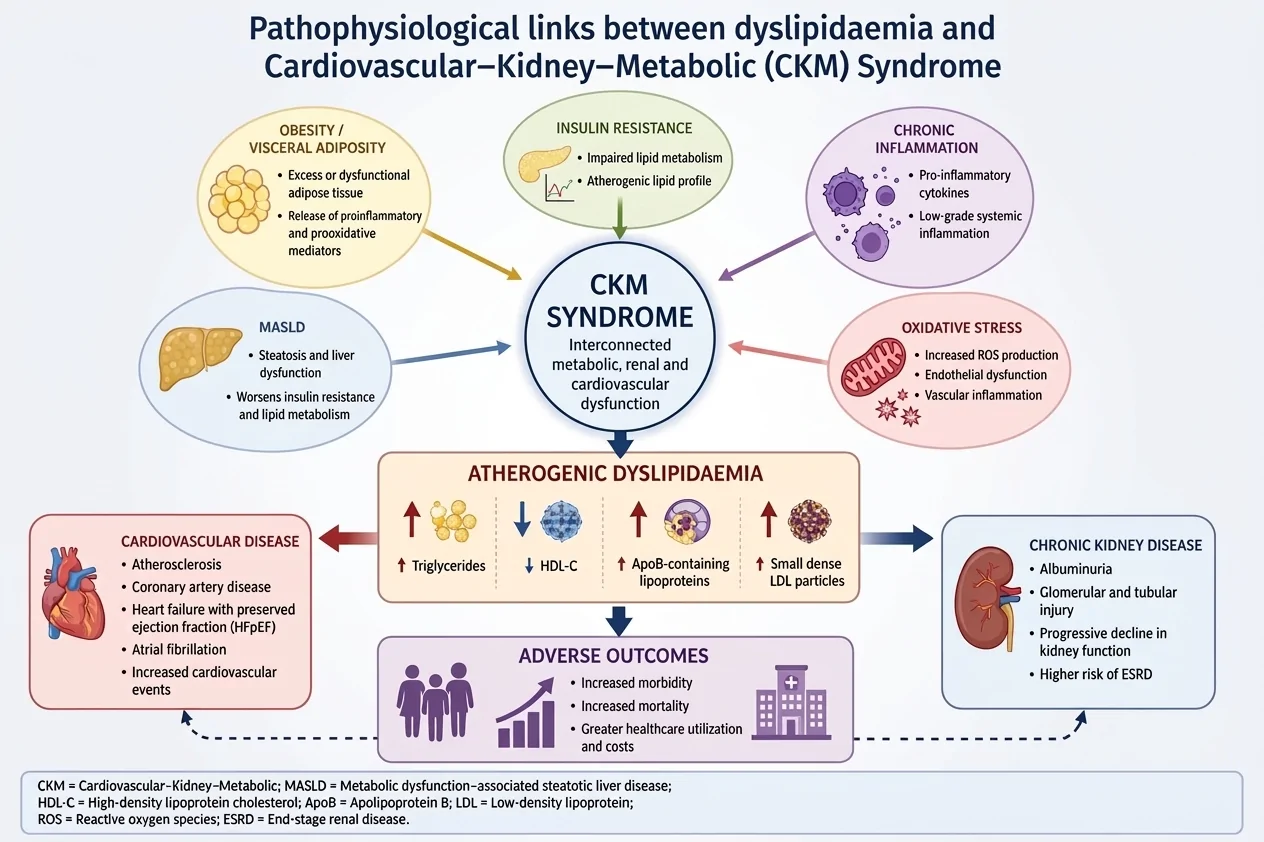
Pathophysiological links between dyslipidaemia and cardiovascular–kidney–metabolic (CKM) syndrome. CKM syndrome arises from a complex interplay of visceral adiposity, insulin resistance, chronic inflammation, metabolic dysfunction-associated steatotic liver disease (MASLD), and oxidative stress. These mechanisms promote the development of atherogenic dyslipidaemia, characterised by elevated triglycerides, reduced HDL cholesterol, increased concentrations of apolipoprotein B-containing lipoproteins, and a predominance of small dense LDL particles. In turn, dyslipidaemia contributes to the progression of cardiovascular disease and chronic kidney disease, ultimately leading to increased morbidity, mortality, and healthcare burden. Created in BioRender. Ceasovschih, A. (2026) https://BioRender.com/6785ham.

Thus, lipid abnormalities in CKM syndrome extend beyond the classical atherogenic lipid profile. The characteristic lipid phenotype, together with ectopic fat accumulation, lipotoxicity, systemic inflammation and oxidative stress, forms a unique pathophysiological profile affecting multiple organ systems (Figure 1). These interconnected mechanisms ultimately translate into clinical manifestations across the cardiovascular and kidney axes of CKM.

## 3. Clinical Implications

Lipid disorders are closely linked to the development and progression of CKM syndrome. Beyond their established role in atherosclerotic cardiovascular disease, lipid abnormalities are associated with renal dysfunction, systemic inflammation, and metabolic deterioration. The coexistence of obesity, insulin resistance, CKD, and dyslipidaemia creates a vicious cycle in which metabolic and vascular abnormalities accelerate damage across multiple organ systems. Consequently, dyslipidaemia should be considered not only a cardiovascular risk factor but also an important component of the broader pathophysiology of CKM syndrome [87,88].

### 3.1. Cardiovascular Risk

Patients with CKM syndrome exhibit a markedly elevated risk of cardiovascular morbidity and mortality. Atherogenic dyslipidaemia promotes the retention of apoB-containing lipoproteins within the arterial wall. Subsequent oxidative modification of LDL particles triggers inflammatory responses, recruitment of macrophages, and formation of foam cells, leading to progressive atherosclerotic plaque development. Insulin resistance further increases hepatic VLDL production and impairs lipoprotein clearance, thereby increasing circulating concentrations of atherogenic lipoproteins [89,90].

The cardiovascular burden is amplified in patients with CKD. Even mild reductions in estimated glomerular filtration rate (eGFR) are associated with increased risks of myocardial infarction, stroke, heart failure (HF), and cardiovascular death [91]. Indeed, CKD is accompanied by profound disturbances in lipid metabolism, including accumulation of TG-rich remnants and altered HDL functionality, which may accelerate atherosclerosis [92].

Emerging evidence suggests that cardiovascular risk in CKM syndrome extends beyond traditional atherosclerotic mechanisms. Lipotoxicity, ectopic fat deposition, and chronic low-grade inflammation adversely affect myocardial structure and function. Excess circulating free fatty acids may accumulate within cardiomyocytes, leading to mitochondrial dysfunction, oxidative stress, and myocardial fibrosis [93]. These processes contribute to the development of HFpEF, a common cardiovascular manifestation among patients with obesity, diabetes, and CKD [94].

Recent studies have highlighted the importance of an integrated risk assessment within the CKM framework. The American Heart Association recognises CKM syndrome as a continuum between metabolic dysfunction, kidney disease, and cardiovascular pathology, emphasising the need for early identification and management of lipid abnormalities to reduce long-term cardiovascular risk. Accordingly, five temporal and clinical stages are recognised: the absence of risk factors (Stage 0), the presence of excess or dysfunctional adiposity including overweight, obesity, or prediabetes (Stage 1), the emergence of metabolic abnormalities or CKD (Stage 2), the development of subclinical cardiovascular disease or very high predicted cardiovascular risk (Stage 3), and overt clinical cardiovascular disease, with Stage 4a in the absence of kidney failure and Stage 4b in the presence of kidney failure [87,88]. Lipid abnormalities evolving across this continuum are reported in Table 1.

Recent advances in cardiovascular risk assessment further support the clinical relevance of the CKM framework. The latest AHA/ACC recommendations have incorporated the PREVENT risk equations, representing the first major update of cardiovascular risk prediction since the 2018 cholesterol guidelines. Unlike previous prediction models, PREVENT integrates chronic kidney disease and body mass index into cardiovascular risk estimation, better reflecting the cardiometabolic factors that characterise CKM syndrome. In addition, the updated recommendations redefine cardiovascular risk categories and emphasise individualised treatment thresholds according to the overall burden of metabolic, renal, and cardiovascular risk factors rather than LDL levels alone. These developments further support a more comprehensive risk assessment and reinforce the importance of early identification and management of dyslipidaemia within an integrated CKM prevention strategy. The incorporation of obesity and kidney dysfunction into contemporary cardiovascular risk prediction models highlights the transition from traditional atherosclerotic cardiovascular disease-centred prevention toward a multidimensional CKM-based approach to cardiovascular risk stratification [1].

### 3.2. Kidney Disease Progression

Dyslipidaemia has been proposed to contribute to the initiation and progression of CKD. Experimental and clinical evidence supports the concept of lipid nephrotoxicity, whereby excessive exposure of renal tissues to circulating lipids promotes structural and functional damage [95].

Glomerular injury is an important manifestation of lipid-mediated renal damage. Elevated concentrations of LDL-C, oxidised LDL, and TG-rich lipoproteins induce endothelial dysfunction within glomerular capillaries and promote mesangial cell activation [95]. Oxidised lipoproteins stimulate inflammatory signalling pathways, leading to increased expression of cytokines, chemokines, and adhesion molecules that contribute to glomerulosclerosis [96].

Mesangial expansion represents another key mechanism linking dyslipidaemia with renal deterioration. Lipid accumulation within mesangial cells promotes extracellular matrix production and cellular proliferation, resulting in progressive glomerular remodelling and loss of filtration capacity [95]. These changes are particularly pronounced in patients with diabetes and metabolic syndrome, where insulin resistance and hyperglycaemia further accelerate renal injury [97].

Tubulointerstitial inflammation is increasingly recognised as a major determinant of CKD progression. Excess fatty acids and lipid metabolites may accumulate within tubular epithelial cells, causing mitochondrial dysfunction, oxidative stress, and apoptosis [98]. This process triggers inflammatory responses and promotes fibrotic remodelling of the renal interstitium. Chronic activation of inflammatory pathways ultimately leads to irreversible nephron loss and progressive decline in kidney function [97].

Moreover, impaired renal function further exacerbates dyslipidaemia through reduced lipoprotein catabolism and altered lipid transport. Thus, CKD and dyslipidaemia are closely interrelated and may reinforce one another through metabolic and renal mechanisms [96,99]. The clinical significance of these interactions is reflected in the markedly increased cardiovascular and renal event rates observed among individuals with CKD [91]. However, whether lipid-lowering therapy can interrupt this cycle and improve renal outcomes remains incompletely established.

### 3.3. Residual Risk Despite LDL-C

Despite substantial reductions in atherosclerotic cardiovascular disease risk achieved with statin therapy, many patients continue to experience cardiovascular events even after achieving guideline-recommended LDL-C targets. This phenomenon, commonly referred to as residual cardiovascular risk, is particularly prominent in individuals with CKM syndrome [100].

Hypertriglyceridaemia represents one of the major contributors to residual risk. Elevated TG-rich lipoproteins and their remnants contain significant amounts of cholesterol capable of penetrating the arterial wall and promoting atherosclerosis. Numerous epidemiological studies have demonstrated independent associations between elevated TG and cardiovascular events, especially in patients with diabetes, obesity, and CKD [101].

Accumulating evidence suggests that RC has a pivotal role in residual cardiovascular risk among patients with CKM syndrome. RC refers to the cholesterol content of TG-rich lipoproteins, including VLDL remnants, IDL, and chylomicron remnants. Unlike TG themselves, remnant particles are capable of penetrating the arterial wall and delivering substantial amounts of cholesterol directly into the intima, thereby promoting foam cell formation and atherosclerotic plaque progression. Recent epidemiological and genetic studies indicate that RC contributes independently to ASCVD risk even in patients who achieve recommended LDL-C targets, suggesting that it represents an important component of residual cardiovascular risk [89,101].

Persistent inflammation is another important driver of residual risk. Even after effective LDL-C lowering, inflammatory activity within atherosclerotic plaques may continue to promote plaque instability and thrombosis [100,102]. High-sensitivity CRP remains a useful marker of residual inflammatory risk, and clinical trials have confirmed that targeting inflammation can further reduce cardiovascular events independent of lipid lowering [102].

Lp(a) has progressively emerged as a particularly important residual risk factor. Lp(a) is a genetically determined lipoprotein particle that possesses both atherogenic and prothrombotic properties [103]. Elevated Lp(a) concentrations are associated with increased risks of myocardial infarction, ischaemic stroke, aortic valve stenosis, and cardiovascular mortality [89]. Importantly, conventional lipid-lowering therapies have limited effects on Lp(a) concentrations, highlighting the need for novel therapeutic approaches. Several therapies designed to specifically reduce Lp(a) levels are currently undergoing clinical evaluation and may provide future opportunities to address Lp(a)-mediated residual cardiovascular risk [104]. Recent evidence suggests that elevated Lp(a) may represent a major contributor to residual cardiovascular risk within the CKM syndrome spectrum [105]. This observation further supports routine assessment of Lp(a) concentrations as part of comprehensive cardiovascular risk stratification.

Recognition of residual risk is particularly relevant in CKM syndrome, where multiple pathophysiological pathways coexist. Comprehensive risk reduction therefore requires not only LDL-C lowering but also management of hypertriglyceridaemia, inflammation, obesity, insulin resistance, kidney dysfunction, and elevated Lp(a) concentrations [88,105].

## 4. Lipid-Lowering Therapeutic Strategies

Taken together, current evidence supports a pragmatic, stepwise approach to dyslipidaemia in CKM syndrome. High-intensity statin therapy remains the first-line treatment for LDL-C lowering in most patients, with ezetimibe added when LDL-C or non-HDL-C targets are not achieved. PCSK9 inhibitors and, in selected patients, bempedoic acid provide additional LDL-C and non-HDL-C reduction when treatment goals remain unmet or when statin intolerance limits therapy. In patients with persistent hypertriglyceridaemia and elevated RC despite statin therapy, icosapent ethyl and, where appropriate, fibrates may be considered to target TG-rich lipoproteins. For patients with elevated Lp(a), selective Lp(a)-lowering therapies are emerging, although cardiovascular outcome data are not yet available.

Lipid-lowering therapies differ not only in their principal lipid target but also in the strength of evidence supporting reductions in hard cardiovascular endpoints. For clarity, these therapies can be broadly distinguished into six categories: (i) LDL-C-lowering therapies with proven effects on cardiovascular outcomes in adequately powered randomised trials, such as statins [106], ezetimibe [107], bempedoic acid in statin-intolerant patients [108] and monoclonal antibodies targeting PCSK9 [109,110]; (ii) LDL-C-lowering therapies approved on the basis of biomarker reduction, for which cardiovascular outcome data remain pending, such as inclisiran [111,112]; (iii) therapies targeting triglycerides and remnant-related residual risk, including icosapent ethyl, which has demonstrated cardiovascular outcome benefit [113] and fibrates, for which outcome evidence has been limited and largely restricted to specific subgroups [114]; (iv) therapies targeting severe hypertriglyceridaemia, primarily to reduce the risk of acute pancreatitis, including apoC-III inhibitors [115,116] and, the ANGPTL3 inhibitor evinacumab, which is approved for homozygous familial hypercholesterolaemia rather than pancreatitis prevention [117]; (v) investigational Lp(a)-lowering therapies, for which reductions in Lp(a) have been demonstrated but cardiovascular outcome data remain unavailable [118,119,120]; and (vi) cardiorenal and anti-obesity therapies including GLP-1 receptor agonists and SGLT2 inhibitors, which may exert secondary effects on lipid metabolism while providing cardiovascular and kidney protection through mechanisms beyond lipid-lowering [121,122,123]. This distinction is clinically significant because it separates therapies supported by cardiovascular outcome data from those for which evidence currently rests primarily on changes in lipid biomarkers. This is particularly relevant in CKM syndrome, where kidney dysfunction may further influence the efficacy, pharmacokinetics and safety of lipid-lowering therapies [124].

### 4.1. Statins

**Figure 2:**
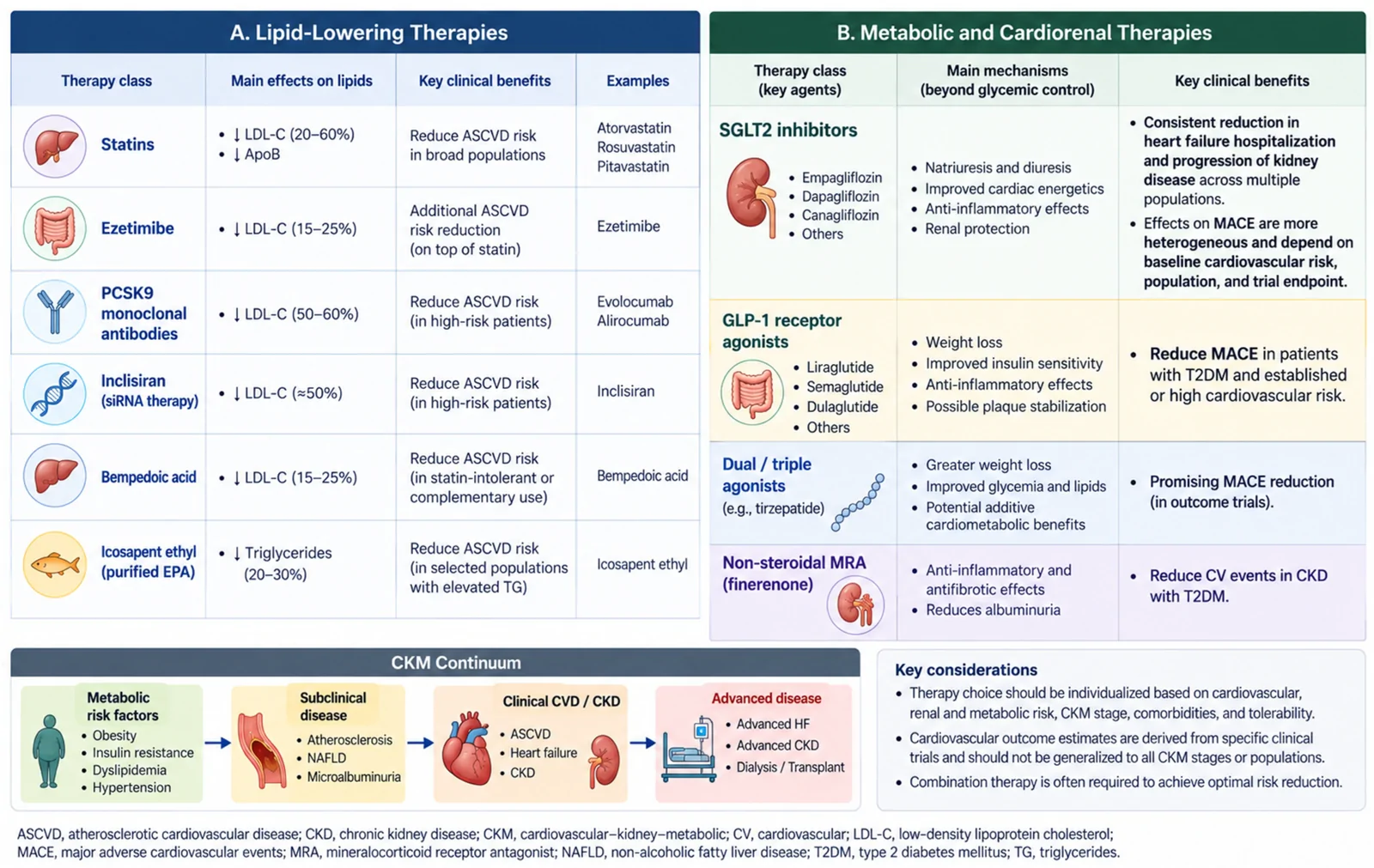
Integrated management approach in cardiovascular–kidney–metabolic (CKM) syndrome. Effective management of CKM syndrome requires a comprehensive, multidisciplinary strategy targeting interconnected metabolic, cardiovascular, and renal pathways. Lifestyle modification, including weight reduction, healthy dietary patterns, regular physical activity, smoking cessation, and sleep optimisation, forms the foundation of care. Pharmacological treatment should address glycaemic control, blood pressure management, renal protection, and dyslipidaemia through the use of evidence-based therapies such as SGLT2 inhibitors, GLP-1 receptor agonists, renin–angiotensin system inhibitors, finerenone, statins, ezetimibe, PCSK9 inhibitors, and triglyceride-lowering agents when indicated. The integration of these interventions across the CKM continuum contributes to reduced cardiovascular events, slower progression of chronic kidney disease, fewer heart failure hospitalisations, and improved long-term survival.

Statins, HMG-CoA reductase inhibitors, are foundational in the management of CKM syndrome. Through their pharmacological effects, they inhibit hepatic cholesterol synthesis, reduce intracellular cholesterol, and upregulate LDL receptors, thereby enhancing the clearance of circulating LDL particles and other apoB-containing lipoproteins. Depending on their potency and dose, statins reduce LDL-C by 30–60% and additionally reduce non-HDL-C [125]. Beyond their lipid effects, statins have been reported to reduce vascular inflammation [126], a pharmacological action potentially relevant in CKM syndrome, where subclinical inflammation is suggested to act as a crucial driver of atherosclerosis progression. Regarding clinical endpoints, statins have demonstrated unequivocal benefit in reducing major adverse cardiovascular events in both secondary and primary prevention [127]. Regardless of the clinical setting, large randomised clinical trials and meta-analyses show that a statin-mediated reduction in LDL-C of 1 mmol/L (39 mg/dL) leads to a decrease of nearly one-fifth in major cardiovascular events [128]. In a meta-analysis of randomised clinical trials and observational cohort studies, the overall prevalence of statin intolerance was estimated to be 9.1% and was mainly attributable to musculoskeletal adverse events (myalgia, myopathy, and rhabdomyolysis) and hepatotoxicity [129]. Nonetheless, leading lipid experts reassure clinicians regarding the use of these drugs as primary lipid-lowering therapy, stressing that their absolute cardiovascular benefit overwhelmingly outweighs these safety risks [130] (Figure 2).

Notably, available evidence shows that in T2DM, statins display comparable benefit, in terms of cardiovascular risk reduction, to that observed in non-diabetic populations [131]. Although there is evidence that statins slightly increase the risk of new-onset diabetes with long-term treatment, this effect is estimated to be modest (≈0.2% per year of treatment, depending on the underlying risk of diabetes mellitus in the population studied) and is clinically outweighed by substantial benefit in cardiovascular event reduction [129]. Furthermore, as demonstrated by the SHARP trial, LDL-C lowering with statins plus ezetimibe reduces cardiovascular events in non-dialysis CKD patients [132], making their role valuable in this clinical setting despite the need for dose adjustment (Table 2). Importantly, however, the cardiovascular benefit of statins attenuates as renal function declines and is largely lost once patients reach dialysis-dependent kidney failure. In the 4D trial, atorvastatin 20 mg did not significantly reduce the composite of cardiac death, myocardial infarction, and stroke in haemodialysis patients with T2DM (HR 0.92, 95% CI 0.77–1.10), despite a 42% reduction in LDL-C [133]. The AURORA trial likewise found no significant effect of rosuvastatin 10 mg on major adverse cardiovascular events (MACE) in a broader haemodialysis population (HR 0.96, 95% CI 0.84–1.11) [134]. Consistent with these results, the benefit observed in SHARP was driven predominantly by non-dialysis participants. This attenuation is thought to reflect a shift in the dominant mechanisms of cardiovascular death in advanced kidney disease, from atherosclerotic events towards arrhythmia, sudden cardiac death, HF, and medial vascular calcification, which are less responsive to LDL-C lowering. Overall, available evidence from randomised clinical trials and large observational studies suggests robust LDL-C-lowering efficacy, cardiovascular protection across different clinical scenarios (including T2DM and CKD), and potential pleiotropic effects of statins, making them the first-line lipid-lowering therapy in CKM syndrome.

### 4.2. Ezetimibe

Ezetimibe, an inhibitor of the Niemann–Pick C1-like 1 (NPC1L1) transporter, plays a central role in contemporary oral lipid-lowering strategies, particularly for patients requiring additional LDL-C and non-HDL-C reduction beyond statins and represents a valuable therapeutic option within CKM syndrome. Indeed, through its mechanism of action, it leads to decreased intestinal cholesterol absorption, reduced hepatic cholesterol stores, upregulation of LDL receptors, and enhanced clearance of circulating apoB-containing lipoproteins. This translates into nearly a 15–20% LDL-C reduction as monotherapy, nearly a 20–25% LDL-C reduction in combination with statins, and a modest non-HDL-C reduction [135] (Figure 2). In the IMPROVE-IT trial, ezetimibe added to statin therapy (simvastatin 40 mg) resulted in a nearly 20% incremental lowering of LDL-C levels and improved cardiovascular outcomes among 18,144 patients who had been hospitalised for an acute coronary syndrome within the preceding 10 days [107]. In the EWTOPIA 75 trial, among 3796 elderly patients aged 75 years or older with elevated LDL-C levels but no history of coronary artery disease, ezetimibe monotherapy showed a significant reduction in the incidence of major adverse cardiovascular events compared with usual care (5.2% versus 7.8%; HR 0.66, 95% CI 0.50–0.86) [136]. Collectively, these findings support the use of ezetimibe as monotherapy or in combination therapy as an effective approach to lowering LDL-C levels and cardiovascular risk in both primary and secondary prevention settings. Ezetimibe has a favourable safety profile based on randomised clinical trials and observational studies, with sporadic reporting of hepatic, musculoskeletal, or gastrointestinal adverse effects [135]. Notably, available evidence suggests that ezetimibe does not worsen glycaemic control, insulin resistance, or body weight, and can therefore be readily integrated into lipid-lowering therapy in the context of CKM syndrome. In addition, ezetimibe may offer a crucial clinical advantage in the management of dyslipidaemia. The SHARP trial demonstrated a significant reduction in major atherosclerotic events in CKD patients receiving the combination of simvastatin 20 mg and ezetimibe 10 mg compared with placebo over a median follow-up of 4.9 years (RR 0.83, 95% CI 0.74–0.94) [132].

### 4.3. Bempedoic Acid

Bempedoic acid, an oral ATP-citrate lyase inhibitor, may be considered increasingly relevant in the integrated management of CKM syndrome, particularly when statins and ezetimibe are insufficient or poorly tolerated. It acts by decreasing hepatic cholesterol synthesis, upregulating LDL receptor expression, and enhancing the clearance of circulating apoB-containing lipoproteins [137]. Therefore, it reduces LDL-C (by approximately 18–25% as monotherapy, and up to 38% when combined with ezetimibe), and, to a lesser extent, non-HDL-C, thereby addressing a large part of the lipoprotein-related atherogenic burden. In addition, it modestly reduces high-sensitivity CRP, thereby also addressing subclinical inflammation [108,138]. In the CLEAR Outcomes trial, bempedoic acid significantly reduced the risk of major adverse cardiovascular events in primary and secondary prevention among 13,970 statin-intolerant patients (composite endpoint of death from cardiovascular causes, nonfatal myocardial infarction, nonfatal stroke, or coronary revascularization occurred in 11.7% vs. 13.3%; HR 0.87, 95% CI 0.79–0.96). According to randomised clinical trials and emerging observational studies, bempedoic acid has a favourable safety profile with no significant increase in musculoskeletal adverse events. However, small increases in uric acid levels, gout attacks, and cholelithiasis have been observed, requiring monitoring in predisposed individuals [108]. Notably, according to subgroup analyses of randomised clinical trials, bempedoic acid consistently lowers LDL-C across glycaemic strata and does not worsen glycaemic variables or increase the incidence of new-onset diabetes versus placebo during long-term treatment [139]. This could be particularly relevant in CKM syndrome, where insulin resistance and abnormalities in glycaemic control amplify cardiovascular risk. In addition, in obese patients, LDL-C lowering efficacy, cardiovascular protection, and the safety profile of bempedoic acid have been reported to be preserved [140] (Figure 2).

This may represent an additional clinical advantage of bempedoic acid in the management of CKM syndrome. Particularly, the limited occurrence of musculoskeletal adverse effects could be advantageous in obese patients given the higher prevalence of statin intolerance in this population as compared to the general population [140]. Furthermore, bempedoic acid maintains its LDL-C-lowering efficacy across moderate CKD (Stage 2 or Stage 3a + b), requires no dose adjustment with declining renal function, and is not associated with an increased risk of adverse events [141].

Additionally, although bempedoic acid treatment has been associated with a modest increase in creatinine levels, there is no evidence of clinically significant renal impairment [124]. For this reason, bempedoic acid could also represent a valuable therapeutic option for the K axis of CKM syndrome (Table 2).

### 4.4. PCSK9 Inhibitors

PCSK9 inhibitors, including currently approved monoclonal antibodies (i.e., alirocumab and evolocumab), inclisiran, and additional developing pharmacological strategies, represent potent and safe lipid-lowering therapies for the management of lipid disorders related to CKM syndrome (Figure 2).

Monoclonal antibodies, by blocking the PCSK9 biological pathway, prevent LDL receptor degradation in hepatocytes, leading to marked reductions in circulating atherogenic lipoproteins. Accordingly, they lower LDL-C by roughly 50–70% on top of statins and/or ezetimibe, and variably reduce non-HDL-C and Lp(a), thereby targeting the full spectrum of apoB-containing particles driving atherosclerosis [142].

Large, randomised clinical trials have shown that alirocumab and evolocumab significantly reduce major adverse cardiovascular events, including myocardial infarction, ischaemic stroke, and coronary revascularisation, with a neutral overall safety profile compared with placebo in patients with documented atherosclerotic cardiovascular disease [143]. In addition, in selected primary-prevention settings, they have been shown to help patients achieve stringent LDL-C targets, preventing first cardiovascular events [144].

Despite some inconsistency, available evidence suggests that anti-PCSK9 monoclonal antibodies significantly lower LDL-C and reduce cardiovascular risk without altering glycaemic control or increasing the risk of developing new-onset diabetes, providing a rationale for using these drugs in patients with disturbances in glucose metabolism [145]. In addition, in obese patients, anti-PCSK9 monoclonal antibodies have been shown to preserve their efficacy and safety [146].

In CKD, anti-PCSK9 monoclonal antibodies have been reported to achieve robust LDL-C lowering without altering serum creatinine levels across a wide range of eGFR values [147]. A pharmacovigilance analysis of the FAERS database reported a lower-than-expected number of acute kidney injury events with PCSK9 inhibitors, generating a hypothesis of potential renal safety (or protection) that requires confirmation in prospective studies [148]. Thus, evolocumab and alirocumab could be particularly valuable in the treatment of dyslipidaemia among CKD patients (Table 2).

Inclisiran represents a first-in-class anti-PCSK9 siRNA-based therapy approved for lowering LDL-C [111]. It acts as a synthetic double-stranded small interfering RNA molecule conjugated with triantennary N-acetylgalactosamine (GalNAc), which facilitates selective uptake by hepatocytes through the asialoglycoprotein receptor [149]. Once internalised into liver cells, inclisiran utilises the endogenous RNA interference pathway to degrade messenger RNA (mRNA) encoding PCSK9. By silencing PCSK9 synthesis, inclisiran increases hepatic LDL receptor density, enhances LDL-C uptake, and produces sustained reductions in circulating LDL-C concentrations [111]. Unlike monoclonal antibodies such as evolocumab and alirocumab, which neutralise circulating PCSK9 protein, inclisiran suppresses PCSK9 production intracellularly, resulting in prolonged therapeutic activity and a unique dosing regimen requiring administration only twice yearly after the initial loading phase [149].

The efficacy and safety of inclisiran have been evaluated in the comprehensive ORION clinical trial programme. The pivotal phase III studies ORION-9, ORION-10, and ORION-11 enrolled patients with heterozygous familial hypercholesterolaemia, established atherosclerotic cardiovascular disease, or atherosclerotic cardiovascular disease-equivalent risk receiving maximally tolerated lipid-lowering therapy. In ORION-10 and ORION-11, inclisiran achieved placebo-adjusted LDL-C reductions of approximately 50%. Importantly, LDL-C lowering was accompanied by significant reductions in non-HDL-C, apoB, and Lp(a), suggesting a broad beneficial effect on the atherogenic lipid profile [111].

One of the major strengths of inclisiran is its favourable safety profile. Across the ORION clinical development programme, adverse event rates were generally comparable to placebo [111]. The most commonly reported adverse events were mild injection-site reactions, which rarely led to treatment discontinuation.

Ongoing cardiovascular outcome trials are evaluating whether inclisiran-mediated LDL-C reduction may translate into reductions in cardiovascular morbidity and mortality [149].

Subgroup analyses of randomised clinical trials and observational studies have demonstrated that inclisiran maintains its lipid-lowering efficacy across different clinical settings, including T2DM, obesity and CKD [150,151,152].

Furthermore, no significant increases in renal dysfunction or new-onset diabetes have been observed during follow-up among patients treated with inclisiran [149]. Thus, across the CKM spectrum, inclisiran may also provide potent and durable atherogenic lipoprotein lowering with a reassuring safety profile (Table 2). Thus, inclisiran may serve as an adjunctive therapy on top of statins and/or ezetimibe among patients with CKM syndrome. Nonetheless, the current cost and accessibility of PCSK9-targeting therapies continue to limit their use in clinical practice.

### 4.5. Therapies Targeting Triglycerides

Hypotriglyceridemic drugs, including fibrates, omega-3 fatty acids (especially icosapent ethyl), and emerging agents targeting apoC-III and ANGPTL3, are particularly relevant to the management of CKM syndrome (Figure 2).

Fibrates activate PPAR-α, enhancing hepatic fatty acid oxidation, reducing VLDL production, and increasing lipoprotein lipase activity, thereby lowering TGs (20–50%) and non-HDL-C and modestly raising HDL-C. Omega-3 fatty acids reduce hepatic VLDL synthesis and secretion, with icosapent ethyl (pure EPA) showing robust reductions in TGs (20–50%) and non-HDL-C. Novel apoC-III and ANGPTL3 inhibitors promote the clearance of TG-rich lipoproteins by directly targeting pathways that regulate lipolysis, resulting in substantial reductions in TG levels (>50%) [153].

In terms of cardiovascular prevention, traditional fibrates have shown modest and often subgroup-restricted benefits, particularly in patients with high TGs and low HDL-C, with overall neutral or limited impact in broad populations [153]. In contrast, icosapent ethyl has demonstrated substantial reductions in major adverse cardiovascular events in high-risk patients with elevated TGs on statins, supporting its use in secondary prevention and selected high-risk primary-prevention settings [113]. The long-term cardiovascular effects of emerging apoC-III and ANGPTL3 inhibitors remain under investigation.

The safety profiles of both fibrates and icosapent ethyl are generally acceptable, though fibrates may increase the risk of myopathy when combined with statins, while omega-3 fatty acids may slightly increase the risk of atrial fibrillation or bleeding under certain conditions [153,154]. The safety profile of emerging apoC-III and ANGPTL3 inhibitors differs significantly depending on the pharmacological platform, with small interfering RNA (siRNA) agents generally better tolerated than ASOs.

In T2DM and obesity, despite targeting atherogenic dyslipidaemia, fibrates do not consistently reduce hard cardiovascular outcomes [153]. Conversely, icosapent ethyl can provide significant cardiovascular risk reduction in diabetic patients without worsening glycaemic control or body weight. This supports the use of icosapent ethyl over fibrates for secondary prevention in diabetic patients [113]. Available clinical studies suggest that emerging apoC-III and ANGPTL3 inhibitors may improve insulin sensitivity, although confirmatory evidence is awaited [155].

In CKD patients, the efficacy of fibrates is preserved, but their use requires caution because of potential increases in serum creatinine and an increased risk of adverse effects [156]. Icosapent ethyl appears relatively safe across a wide range of kidney function and maintains cardiovascular benefit in CKD subgroups, making it an attractive option in this setting for reducing residual cardiovascular risk driven by elevated TGs [157]. Data on emerging apoC-III and ANGPTL3 inhibitors in CKD are still scarce, but their mechanism of action may be particularly relevant to the K axis of CKM syndrome. Overall, within CKM syndrome, available hypotriglyceridemic drugs can provide targeted reductions in TG-rich lipoproteins, potentially offering incremental cardiovascular and metabolic benefit when carefully selected and monitored. However, among them, only fibrates and icosapent ethyl can currently be prescribed for mild to moderate hypertriglyceridaemia, potentially offering more widely applicable options for managing cardiometabolic risk (Table 2). Instead, APOC-III and ANGPTL3 inhibitors are currently approved only for niche indications (i.e., patients with severe hypertriglyceridaemia or patients with familial chylomicronaemia syndrome with TG levels ≥ 1000 mg/dL and a high risk of recurrent acute pancreatitis, and patients with homozygous familial hypercholesterolaemia, respectively) [158].

### 4.6. Therapies Targeting Lp(a)

Until recently, therapeutic options for lowering Lp(a) were limited. Conventional lipid-lowering therapies, including statins, have little effect on Lp(a) concentrations and may even slightly increase them. Ezetimibe produces minimal reductions in Lp(a), while PCSK9 inhibitors lower Lp(a) by only 20–30%, which may be insufficient in patients with markedly elevated baseline levels [159]. Lipoprotein apheresis remains effective but is invasive, costly, and available only in selected centres. These limitations have stimulated the development of targeted therapies aimed at suppressing hepatic Lp(a) production (Figure 2).

Pelacarsen (formerly AKCEA-APO(a)-LRx) is the first antisense oligonucleotide specifically designed to target hepatic apo(a) mRNA. By binding to apo(a) mRNA within hepatocytes, pelacarsen promotes its degradation through RNase H-mediated cleavage, thereby reducing apo(a) synthesis and circulating Lp(a) concentrations [160]. In a phase II randomised clinical trial involving patients with established cardiovascular disease and elevated Lp(a), pelacarsen achieved dose-dependent reductions in Lp(a) levels ranging from 35% to over 80%. At the highest doses, reductions exceeded 80% [161]. These promising results led to the initiation of the landmark Lp(a)HORIZON trial, a phase III cardiovascular outcomes study evaluating whether Lp(a) lowering with pelacarsen reduces major adverse cardiovascular events in patients with established ASCVD and elevated Lp(a) concentrations [162]. However, in September 2026, Novartis announced that the phase III Lp(a)HORIZON trial (n = 8323) did not meet its primary endpoint. Pelacarsen, despite substantially lowering Lp(a) levels, did not reduce the risk of the composite endpoint of cardiovascular death, non-fatal myocardial infarction, non-fatal stroke, and urgent coronary revascularization requiring hospitalisation compared with placebo.

In parallel, several siRNA-based therapies targeting Lp(a) synthesis have entered clinical development. These agents exploit the endogenous RNA interference pathway to silence hepatic apo(a) production, producing potent and durable reductions in circulating Lp(a).

Olpasiran is a GalNAc-conjugated siRNA that selectively inhibits apo(a) synthesis in hepatocytes. The phase II OCEAN(a)-DOSE trial demonstrated reductions in Lp(a) concentrations of more than 90% among patients receiving higher-dose regimens. Notably, these reductions were sustained for extended periods after dosing, suggesting the possibility of infrequent administration schedules and improved patient adherence. Olpasiran was generally well tolerated, with injection-site reactions representing the most common adverse event [118].

Lepodisiran is another investigational siRNA therapy targeting apo(a) synthesis. Early clinical studies have demonstrated efficacy, with single-dose administration producing reductions in Lp(a) exceeding 90%, which persisted for many months after treatment [163].

Additional therapies, including the siRNA agent zerlasiran and the oral small-molecule inhibitor muvalaplin, are currently being investigated. These agents may further expand the therapeutic landscape for Lp(a) reduction and reflect growing recognition of Lp(a) as a major modifiable cardiovascular risk factor [164].

Overall, the development of selective therapies targeting Lp(a) may represent a major advance in preventive cardiology in recent decades. Unlike traditional lipid-lowering agents, these therapies specifically address a genetically mediated cardiovascular risk factor that has historically been difficult to modify. Current evidence suggests that circulating Lp(a) concentrations can be reduced by 80–95%, substantially exceeding the effects observed with existing therapies [118,163]. However, the ultimate clinical value of these therapies depends on demonstrating corresponding reductions in cardiovascular morbidity and mortality. Ongoing large-scale cardiovascular outcome trials with these therapies are expected to provide definitive evidence regarding the clinical benefits of intensive Lp(a) lowering for cardiovascular prevention among patients with markedly elevated Lp(a) concentrations [119]. In addition, these studies and their subgroup analyses may clarify the role of targeting Lp(a) in the context of the CKM syndrome (Table 2).

## 5. Other Therapeutic Strategies

Across all CKM stages, additional therapeutic strategies beyond lipid-lowering therapies are crucial for improving cardiometabolic and renal risk. In particular, a thorough multi-targeted approach that addresses body weight, glucose dysregulation, and blood pressure beyond lipid abnormalities seems essential to offer complementary benefits in CKM syndrome. The basis for such a comprehensive approach arises from several fundamental principles:

- *Common Pathophysiology and Bidirectional Communication within Different Organs.* Various pathophysiological mechanisms are prevalent throughout the CKM spectrum, encompassing neurohormonal dysregulation (notably the activation of the renin–angiotensin–aldosterone system), insulin resistance linked to visceral obesity, oxidative stress, systemic inflammation, and endothelial dysfunction. These interconnected processes establish self-perpetuating cycles of multi-organ damage, wherein each element further promotes the progression of the others [3,165].
- *Aggregated Risk Associated with Multimorbidity.* The intersection of metabolic, renal, and cardiovascular disorders collectively increases patient risk relative to isolated conditions. Each stage transition in CKM syndrome results in an increasingly elevated risk of morbidity and mortality, with an increase in hospital readmissions and medical expenses [8].
- *Accessibility of Multisystem Therapies.* An increasing array of available and emerging treatment approaches positively influences metabolic risk factors, renal function, or both, while also offering cardioprotection, rendering combined management both viable and potentially highly effective [166].

### 5.1. Lifestyle Interventions

A comprehensive lifestyle intervention has been shown to have substantial benefits across all stages of CKM syndrome, in terms of improved cardiovascular and renal outcomes as well as improved quality of life and prolonged survival. In addition, lifestyle modifications may enhance the effectiveness of pharmacological therapies targeting CKM syndrome [167].

The benefits of lifestyle intervention are largely mediated through improvements in dietary habits and physical activity, both of which exert favourable effects on multiple components of the CKM continuum.

Dietary modification remains one of the most effective non-pharmacological approaches to CKM syndrome. Numerous epidemiological studies and randomised clinical trials have demonstrated a strong association between different dietary patterns and cardiovascular outcomes. Among the various dietary patterns, the Mediterranean diet, due to its content of fibre, monounsaturated and omega-3 polyunsaturated fatty acids, and polyphenols, has accumulated the strongest evidence for cardiovascular protection. The landmark PREDIMED trial demonstrated that adherence to a Mediterranean diet supplemented with extra-virgin olive oil or nuts significantly reduced the risk of major cardiovascular events compared with a low-fat control diet [168]. The Mediterranean diet favourably modifies the lipid profile by reducing total cholesterol, LDL-C, and TG levels while preserving or modestly increasing HDL-C [169]. Beyond changes in conventional lipid parameters, adherence to the Mediterranean diet also reduces LDL oxidation, decreases the proportion of small dense LDL particles, and improves overall LDL atherogenicity, effects that may substantially contribute to its cardioprotective properties [170]. Finally, the cardioprotective effects of the Mediterranean diet are also attributed to its anti-inflammatory, antioxidant, and endothelial-protective properties. Reduction of sodium intake represents another important dietary intervention for cardiovascular and renal protection. Indeed, excessive sodium consumption (>2 g/day of sodium–approximately 5 g/day of salt) contributes to hypertension, which remains a major risk factor for cardiovascular disease, HF, and CKD [171]. Current guidelines for cardiovascular prevention recommend diets rich in vegetables, fruits, whole grains, legumes, nuts, fish, and unsaturated fats while limiting the intake of saturated fats, trans fats, refined carbohydrates, processed foods, and sodium [167].

Regular physical activity is another powerful lifestyle intervention for cardiovascular health promotion. Meta-analyses have demonstrated inverse relationships between physical activity levels and all-cause mortality, cardiovascular mortality, coronary heart disease, and stroke [172]. Exercise lowers blood pressure, enhances insulin sensitivity, improves the lipid profile, promotes weight control, and reduces systemic inflammation [173,174,175]. Current guidelines for cardiovascular prevention recommend at least 150–300 min of moderate-intensity aerobic physical activity or 75–150 min of vigorous-intensity activity per week, supplemented by resistance training on two or more days weekly [167].

Excess adiposity promotes hypertension, dyslipidaemia, insulin resistance, inflammation, endothelial dysfunction, and thrombosis, all of which accelerate atherosclerosis and cardiovascular risk [176]. The obesity-driven atherogenic dyslipidaemic profile is characterised by elevated TG, reduced HDL-C, increased small dense LDL particles, and increased apoB-containing lipoproteins, primarily driven by insulin resistance, enhanced adipose tissue lipolysis, increased hepatic VLDL production, and impaired TG-rich lipoprotein clearance [177,178]. Weight reduction consistently improves these lipid abnormalities. A systematic review and meta-analysis of 73 studies involving overweight and obese adults demonstrated that each 1 kg of weight loss was associated with reductions of approximately 1.3 mg/dL in TG, 0.8 mg/dL in LDL-C, and 0.5 mg/dL in total cholesterol, whereas HDL-C initially decreased during active weight loss but increased during the weight maintenance phase. Overall, a 5–10% reduction in body weight resulted in clinically meaningful improvements in TG (approximately −16 mg/dL) and LDL-C (approximately −10 mg/dL) [179]. These findings support sustained weight reduction as an effective lifestyle intervention for improving the atherogenic lipid profile and reducing cardiovascular risk. In addition, weight reduction improves multiple other cardiovascular risk factors and modifiers. Even a modest weight loss of 5–10% of initial body weight has been associated with significant improvements in blood pressure, glycaemic control, and inflammatory markers [180]. Comprehensive weight-management strategies should combine dietary modification, physical activity, behavioural interventions, and, when appropriate, pharmacological or surgical treatment [166,181].

Tobacco use increases the risk of coronary artery disease, stroke, peripheral arterial disease, sudden cardiac death, and HF through multiple mechanisms, including endothelial dysfunction, oxidative stress, inflammation, thrombosis, and accelerated atherosclerosis [182]. Current cigarette smoking is associated with a more atherogenic lipid profile characterised by higher total cholesterol, LDL-C, and TG levels together with lower HDL-C [183]. Following smoking cessation, HDL metabolism improves, resulting in an approximately 0.10 mmol/L (3.9 mg/dL) increase in HDL-C without significant changes in total cholesterol, LDL-C, or TG levels [184]. Smoking cessation produces immediate and long-term cardiovascular benefits. Within one year of cessation, the risk of coronary heart disease decreases substantially, while long-term abstinence may reduce cardiovascular risk to levels approaching those of never-smokers [185]. Behavioural counselling, nicotine replacement therapy, varenicline, and bupropion are effective interventions that significantly improve quit rates.

The relationship between alcohol consumption and cardiovascular disease remains complex. While earlier observational studies suggested potential cardiovascular benefits of moderate alcohol intake, more recent evidence indicates that no level of alcohol consumption can be considered completely safe from a health perspective [186]. Alcohol consumption has complex, dose-dependent effects on lipid metabolism. A systematic review and meta-analysis of interventional studies demonstrated that moderate alcohol intake significantly increased HDL-C by approximately 0.094 mmol/L (3.6 mg/dL) in a dose-dependent manner, while exerting no significant effects on LDL-C, total cholesterol, or TG levels [187]. In contrast, excessive alcohol consumption is associated with hypertriglyceridaemia through increased hepatic VLDL synthesis and impaired TG clearance thereby contributing to an atherogenic lipid profile. In addition, excessive alcohol consumption contributes to hypertension, atrial fibrillation, cardiomyopathy, stroke, obesity, and liver disease. Consequently, current cardiovascular prevention guidelines recommend limiting alcohol intake and avoiding excessive consumption [167].

Sleep has emerged as an important determinant of cardiovascular health. Both insufficient sleep (<7 h per night) and excessive sleep duration (>9 h per night) have been associated with increased risk of hypertension, obesity, diabetes, coronary artery disease, stroke, and cardiovascular mortality [188]. Sleep quality is equally important as sleep duration. Poor sleep quality, frequent nocturnal awakenings, fragmented sleep, and circadian rhythm disturbances have been associated with adverse cardiovascular outcomes independent of traditional risk factors [189]. Sleep disorders such as obstructive sleep apnea further contribute to cardiovascular risk through sympathetic activation, intermittent hypoxia, inflammation, and endothelial dysfunction. Recognition and treatment of sleep disorders are increasingly incorporated into comprehensive cardiovascular prevention programmes [188].

Psychological stress, depression, anxiety, and social isolation are increasingly recognised as contributors to cardiovascular disease development and progression. Chronic stress activates neurohormonal pathways, increases inflammatory activity, and adversely affects cardiovascular risk behaviours. Stress-reduction strategies including mindfulness-based interventions, cognitive behavioural therapy, meditation, social support, and structured rehabilitation programmes have demonstrated beneficial effects on cardiovascular health and overall well-being [190].

Overall, lifestyle interventions remain crucial for cardiovascular prevention despite remarkable advances in pharmacological therapies. The integration of personalised nutrition, digital health technologies, wearable devices, behavioural medicine, and precision prevention strategies is expected to further enhance the effectiveness of lifestyle modification programmes. Future cardiovascular care will likely increasingly combine evidence-based lifestyle interventions with targeted pharmacological therapies to achieve optimal cardiovascular risk reduction and improve long-term clinical outcomes [167].

### 5.2. GLP-1 RAs

Glucagon-like peptide-1 receptor agonists (GLP-1 RAs) represent one of the most significant therapeutic advances in cardiology and nephrology over the last decade. Initially developed for the treatment of T2DM, these agents have demonstrated substantial cardiovascular, metabolic, and renal benefits extending well beyond glycaemic control [191,192,193] (Figure 2).

GLP-1 is an endogenous incretin hormone secreted by intestinal L-cells in response to nutrient intake. Physiologically, GLP-1 enhances glucose-dependent insulin secretion, suppresses glucagon release, delays gastric emptying, and promotes satiety. However, native GLP-1 is rapidly degraded by dipeptidyl peptidase-4 (DPP-4), resulting in a very short plasma half-life. GLP-1 RAs were therefore developed as synthetic analogues resistant to DPP-4 degradation, allowing sustained receptor activation and prolonged biological activity [194].

The cardiovascular, metabolic, and renal benefits of GLP-1 RAs appear to result from multiple complementary mechanisms. These agents produce significant reductions in body weight, particularly visceral adiposity, which is closely associated with insulin resistance, dyslipidaemia, hypertension, and systemic inflammation. Weight reduction achieved with GLP-1 RAs frequently exceeds 10–15% of baseline body weight, approaching outcomes previously achievable only through bariatric surgery [195]. In addition to weight loss, GLP-1 RAs improve glycaemic control without substantially increasing the risk of hypoglycaemia. They also reduce systolic blood pressure, improve lipid profiles, decrease oxidative stress, and attenuate vascular inflammation [193]. Experimental studies have demonstrated favourable effects on endothelial function, plaque stabilisation, and myocardial metabolism, suggesting direct cardiovascular protective effects beyond metabolic improvements [196]. Accumulating evidence further indicates that GLP-1 RAs may reduce atherosclerotic progression through anti-inflammatory pathways. Activation of GLP-1 receptors suppresses pro-inflammatory cytokine production, reduces macrophage activation, and improves vascular homeostasis, thereby contributing to a reduction in atherosclerotic plaque vulnerability [197]. Moreover, GLP-1 RAs exert modest but consistent favourable effects on lipid metabolism. In the LEADER trial, liraglutide treatment was associated with modest reductions in total cholesterol, LDL-C, and TG levels compared with placebo, contributing to an overall improvement in the lipid profile. Likewise, in the SUSTAIN-6 trial, semaglutide significantly reduced total cholesterol, LDL-C, and TG levels compared with placebo [191,192]. More recently, a meta-analysis of placebo-controlled randomised trials demonstrated significant GLP-1 RA-mediated reductions in LDL-C (−2.93 mg/dL) and total cholesterol (approximately −7 mg/dL) [198]. Overall, the lipid-lowering effects of GLP-1 RAs are generally modest but may contribute to their overall cardiometabolic benefits [199]. Mechanistically, these agents appear to reduce hepatic VLDL production, suppress intestinal chylomicron secretion, improve insulin sensitivity, and decrease postprandial lipemia [198].

The cardiovascular safety and efficacy of GLP-1 RAs have been extensively evaluated in large randomised clinical trials. In the landmark LEADER trial, liraglutide significantly reduced the incidence of major cardiovascular events, including cardiovascular death, nonfatal myocardial infarction, and nonfatal stroke, in more than 9000 patients with T2DM at high cardiovascular risk. Furthermore, significant reductions in cardiovascular mortality and all-cause mortality were observed. Similarly, the SUSTAIN-6 trial demonstrated that semaglutide significantly reduced major cardiovascular events in patients with T2DM and elevated cardiovascular risk [191,192]. The REWIND trial, evaluating dulaglutide, expanded these findings by demonstrating cardiovascular benefit even among patients with lower baseline cardiovascular risk. Indeed, REWIND included a substantial proportion of participants without established cardiovascular disease, suggesting a potential role for GLP-1 RAs in primary prevention strategies [200]. The SELECT trial, enrolling overweight or obese individuals without diabetes but with established cardiovascular disease, demonstrated a significant reduction in major adverse cardiovascular events among patients receiving semaglutide compared with placebo [121]. Moreover, emerging evidence suggests potential benefits of GLP-1 RAs in selected HF populations, particularly among patients with obesity-related HFpEF [201]. Finally, GLP-1 RAs have also demonstrated favourable renal effects, including reductions in albuminuria and slower progression of diabetic kidney disease. The FLOW trial showed that semaglutide significantly reduced the risk of major kidney outcomes, including kidney failure (initiation of dialysis, kidney transplantation, or an eGFR < 15 mL/min/1.73 m^2^), a sustained ≥50% decline in eGFR from baseline, or death from kidney-related or cardiovascular causes [122], further strengthening the overall cardiorenal protective profile of GLP-1 RAs [193].

Overall, GLP-1 RAs currently represent an important therapeutic option in modern preventive cardiology and a major advance in contemporary cardiometabolic medicine [121,193], while future research is expected to further clarify their role in HF and CKD.

### 5.3. SGLT2 Inhibitors

Sodium-glucose cotransporter-2 (SGLT2) inhibitors represent one of the most important therapeutic advances in contemporary cardiometabolic and renal medicine. Initially developed as glucose-lowering agents for patients with T2DM, SGLT2 inhibitors have demonstrated clinically meaningful benefits that extend far beyond glycaemic control. In particular, their role has expanded from diabetology into cardiology and nephrology, in the management of HF and CKD [202,203] (Figure 2).

SGLT2 inhibitors act in the proximal renal tubule by reducing glucose and sodium reabsorption. This results in glucosuria, natriuresis, mild osmotic diuresis, reduction in plasma volume, and favourable effects on blood pressure and cardiac loading conditions. However, their cardiorenal benefits cannot be explained solely by glucose reduction. Proposed mechanisms include improved myocardial energetics, reduction in preload and afterload, decreased intraglomerular pressure, attenuation of inflammation and oxidative stress, improved endothelial function, and slowing of renal fibrosis [204,205].

The first major evidence supporting the cardiovascular benefit of these drugs came from the EMPA-REG OUTCOME trial, in which empagliflozin significantly reduced cardiovascular mortality and hospitalisation for HF in patients with T2DM and established cardiovascular disease [202]. Subsequently, the DAPA-HF trial showed that dapagliflozin reduced worsening HF and cardiovascular death in patients with HF with reduced ejection fraction (HFrEF), regardless of diabetes status [203]. Similar benefits were confirmed with empagliflozin in the EMPEROR-Reduced trial [206]. Importantly, SGLT2 inhibitors have also demonstrated efficacy in HF with mildly reduced and preserved ejection fraction (HFmrEF and HFpEF). The EMPEROR-Preserved and DELIVER trials confirmed reductions in HF hospitalisation across a broad spectrum of left ventricular ejection fraction [207,208].

Beyond HF, SGLT2 inhibitors have transformed nephrology. In DAPA-CKD, dapagliflozin significantly reduced the risk of sustained decline in kidney function, end-stage kidney disease, or death from renal or cardiovascular causes in patients with CKD, including those without diabetes [209]. EMPA-KIDNEY further confirmed renal protection with empagliflozin across a wide spectrum of CKD patients [123].

Overall, from a clinical perspective, SGLT2 inhibitors have become a foundational therapy in patients with HF and CKD, irrespective of diabetes status. Their benefits are rapid, consistent, and largely independent of glycaemic status, making them true cardiorenal protective agents rather than merely antidiabetic drugs.

### 5.4. Blood Pressure Control

Blood pressure control represents another fundamental component of CKM syndrome management, given the strong association between hypertension and both cardiovascular and renal disease progression as well as the reduction in cardiovascular and renal risk associated with improved blood pressure control [166].

Antihypertensive pharmacological therapy is recommended for patients with diabetes, CKD, age ≥65 years, or a predicted 10-year cardiovascular risk of ≥10%, in addition to lifestyle modification. The guidelines recommend a blood pressure target of <130/80 mm Hg for all patients with CKM syndrome [210].

ACE inhibitors and angiotensin receptor blockers (ARBs) are the preferred antihypertensive medicines for patients with CKM syndrome, especially those with diabetes, albuminuria, or CKD, as they have been proven to mitigate the progression of renal disease and reduce adverse cardiovascular events. Indeed, in addition to regulating blood pressure, suppression of the renin–angiotensin system provides cardiovascular and renal protection [210].

Furthermore, the nonsteroidal mineralocorticoid receptor antagonist finerenone has shown a significant decrease in the progression of kidney disease, incidence of cardiovascular events, and hospitalisations due to HF in patients with T2DM and CKD who are undergoing optimised ACE inhibitor or ARB therapy [211].

## 6. Future Directions

Future research priorities in lipid disorders associated with CKM syndrome should concentrate on four interrelated areas: (1) enhancing lipid phenotype characterisation, (2) discovering precision biomarkers, (3) assessing emerging therapeutics, and (4) formulating innovative strategies for residual risk mitigation. These research avenues may address key evidence gaps that hinder effective cardiovascular and renal risk reduction in this high-risk demographic.

A prevalent lipid phenotype in CKD, characterised by increased TGs, TGRLs (VLDL particles), small LDL particles, and diminished HDL particles, exhibits a more robust correlation with atherosclerotic cardiovascular disease in CKD patients than in those without CKD. Future research must comprehensively delineate the evolution of lipid abnormalities across the CKM spectrum, from initial metabolic dysfunction to advanced kidney disease, and identify the specific lipoprotein alterations that influence cardiovascular and renal outcomes. Determining whether specific phenotypes predict different therapeutic responses could facilitate personalised treatment strategies [212,213].

Interest in novel biomarkers that may more accurately predict cardiovascular and renal outcomes has been sparked by the apparent limited prognostic value of traditional lipid markers, particularly LDL-C, in patients with CKM syndrome. New evidence indicates that RC, apoB, Lp(a), and non-conventional lipid indices, including the non-HDL-to-HDL cholesterol ratio, the natural logarithm of RC, and the cholesterol-HDL-glucose index, are more strongly associated with atherosclerotic cardiovascular disease and kidney function decline. These biomarkers have the potential to enhance risk stratification and enable a more personalised approach to CKM management. Future research should concentrate on the validation of their clinical utility, the establishment of stage-specific thresholds, and the determination of whether biomarker-guided therapeutic strategies may enhance long-term cardiovascular and renal outcomes [214,215,216].

The persistent challenge of residual cardiovascular risk in CKM syndrome is underscored by the fact that a significant number of patients continue to experience cardiovascular events, despite intensive LDL-C lowering. As a result, the focus of emerging therapeutic strategies should also shift towards targeting non-LDL lipid fractions and additional pathogenic mechanisms contributing to this residual risk. Furthermore, future research in CKM syndrome lipid management should also address the substantial evidence-to-practice gap in current lipid management. Indeed, despite the availability of highly effective lipid-lowering therapies, dyslipidaemia remains substantially undertreated in routine clinical practice [217,218].

Several promising associations and mechanisms discussed in this review, as well as new biomarkers, are currently supported by experimental data, observational data, or surrogate endpoint data. Their implementation in clinical practice will require validation through high-quality randomised trials that can demonstrate a reduction in severe cardiovascular or renal outcomes.

## 7. Conclusions

Dyslipidaemia is both an epiphenomenon and a driver of the intricate pathophysiological interactions characterising CKM syndrome. Lipid metabolism derangements in CKM syndrome represent a heterogeneous constellation of abnormalities that require a more granular and phenotype-driven approach than traditional LDL-centric management alone. Thus, for instance, apoB, non-HDL-C, and Lp(a) can provide incremental value beyond LDL-C to quantify the atherogenic burden more accurately and refine residual-risk assessment across the CKM continuum. In addition, beyond outcome-proven LDL-C-lowering therapies (including statins, ezetimibe, bempedoic acid, and PCSK9 inhibitors), other lipid-lowering therapies targeting the broad spectrum of apoB-containing lipoproteins (including selective Lp(a) lowering therapies, apoC-III inhibitors, and ANGPTL3 inhibitors), although investigational and without completed outcome trials, should be considered as potential future therapeutic options.

It is important to distinguish between mechanistic and observational evidence supporting a role for dyslipidaemia in renal injury and evidence that lipid-lowering therapy improves renal outcomes. While experimental models and observational studies consistently demonstrate lipid-mediated renal damage, randomised clinical trials of lipid-lowering therapy have not consistently shown significant slowing of CKD progression. Thus, while the biological rationale for lipid nephrotoxicity is convincing, the clinical benefit of lipid-lowering therapy in slowing CKD progression remains to be established.

Strengthening collaboration between primary care and specialist lipid services will be essential to ensure timely identification, appropriate counselling, and coordinated therapeutic implementation in patients with CKM syndrome as the therapeutic landscape continues to evolve. Future investigations centred on precision medicine, innovative biomarkers, and targeted therapy strategies will be essential to explore the potential for improving cardiovascular and renal outcomes throughout the CKM continuum.

## Figures and Tables

**Table 1:** Lipid abnormalities and treatment strategies across the CKM continuum.

	CKM Stage 0	CKM Stage 1	CKM Stage 2	CKM Stage 3	CKM Stage 4a	CKM Stage 4b
Typical lipid features	Generally normal lipid profile; no characteristic CKM-related dyslipidaemia	Often normal lipid profile; early increases in TG and/or apoB and decreases in HDL-C may accompany excess adiposity and insulin resistance	Atherogenic dyslipidaemia may include elevated TG, apoB and non-HDL-C; remnant accumulation may occur, particularly in patients with CKD	Persistent atherogenic lipoprotein burden may include elevated apoB, non-HDL-C and remnant cholesterol; phenotype may be modified by CKD	Atherogenic lipoprotein burden is common; phenotype varies with ASCVD, diabetes, obesity and CKD status	Kidney failure-associated lipoprotein remodelling; phenotype varies with dialysis status
Lipid measurements	TC, LDL-C, HDL-C, TG, non-HDL-C, Lp(a) once; apoB optional	TC, LDL-C, HDL-C, TG, non-HDL-C, RC, Lp(a) once, apoB	TC, LDL-C, HDL-C, TG, non-HDL-C, RC, Lp(a) once, apoB	TC, LDL-C, HDL-C, TG, non-HDL-C, RC, Lp(a) once, apoB	TC, LDL-C, HDL-C, TG, non-HDL-C, RC, Lp(a) once, apoB	TC, LDL-C, HDL-C, TG, non-HDL-C, RC, Lp(a) once, apoB; interpret LDL-C in context of advanced CKD
Treatment strategies	Lifestyle intervention; pharmacological lipid lowering only if independently indicated (e.g., genetic dyslipidaemia)	Lifestyle intervention; management of excess adiposity and metabolic risk factors; lipid-lowering therapy according to LDL-C level and ASCVD risk	Lifestyle intervention; management of diabetes, hypertension, and CKD progression; initiate/intensify LDL-C-lowering therapy according to ASCVD risk, CKD status and LDL-C level	Lifestyle intervention; management of diabetes, hypertension, and CKD progression; statins + additional lipid-lowering drugs if needed (e.g., icosapent ethyl for residual risk)	High-intensity statin for clinical ASCVD; add ezetimibe and/or PCSK9-directed therapy as needed to achieve guideline-based LDL-C targets; aggressive management of CKM comorbidities	In dialysis-dependent kidney failure, do not routinely initiate statin or statin/ezetimibe; continue pre-existing therapy when appropriate. In non-dialysis kidney failure, individualise lipid-lowering therapy according to ASCVD status and overall risk

**Table 2:** CKD-specific framing of lipid-lowering therapies.

Therapy	CKD G1–G2	CKD G3–G5, Non-Dialysis	Dialysis-Dependent CKD
Statins	Dose adjustment: Usually not required; check the specific statin. CV outcome evidence: Established in indicated populations. Safety: Myopathy, liver enzyme elevations and modest diabetes risk.	Dose adjustment: Drug-specific; dose modification may be required in severe CKD (eGFR < 30), depending on statin. CV outcome evidence: Established in non-dialysis CKD. Safety: Myopathy, hepatotoxicity and modest diabetes risk.	Dose adjustment: Drug-specific. CV outcome evidence: Benefit attenuated and largely lost in dialysis-dependent kidney failure; routine initiation is not supported. Safety: Myopathy, hepatotoxicity and modest diabetes risk.
Ezetimibe	Dose adjustment: Not required. CV outcome evidence: Established, particularly with a statin. Safety: Generally well tolerated; liver enzyme elevations or myopathy may occur, particularly with a statin.	Dose adjustment: Not required for ezetimibe; check the coadministered statin. CV outcome evidence: Demonstrated for simvastatin plus ezetimibe in SHARP, with benefit driven predominantly by non-dialysis participants. Safety: Generally well tolerated; consider the safety of combination therapy.	Dose adjustment: Not required for ezetimibe. CV outcome evidence: Insufficient evidence/data for a clear dialysis-specific benefit. Safety: Generally well tolerated; consider the safety of a coadministered statin.
Bempedoic acid	Dose adjustment: Not required. CV outcome evidence: Established in statin-intolerant patients. Safety: Hyperuricaemia, gout and cholelithiasis.	Dose adjustment: No adjustment generally required with declining renal function; clinical data are strongest in moderate CKD. CV outcome evidence: Cardiovascular benefit established in statin-intolerant high-risk patients; advanced CKD-specific outcome data remain limited. Safety: Hyperuricaemia, gout, cholelithiasis and modest creatinine increases.	Dose adjustment: Insufficient evidence/data. CV outcome evidence: Insufficient evidence/data. Safety: Insufficient evidence/data.
Evolocumab	Dose adjustment: Not required. CV outcome evidence: Established. Safety: Injection-site reactions; generally well tolerated.	Dose adjustment: Not required. CV outcome evidence: LDL-C lowering and cardiovascular efficacy were consistent across studied CKD groups in FOURIER; data in more advanced CKD remain limited. Safety: Generally well tolerated in studied CKD groups.	Dose adjustment: No adjustment generally required based on available pharmacokinetic data. CV outcome evidence: Insufficient evidence/data. Safety: Limited dialysis-specific data.
Alirocumab	Dose adjustment: Not required. CV outcome evidence: Established. Safety: Injection-site reactions; generally well tolerated.	Dose adjustment: Not required in mild/moderate impairment; severe impairment data are limited. CV outcome evidence: Cardiovascular benefit established overall, with less certain evidence in advanced CKD. Safety: Generally well tolerated in studied groups; advanced CKD data are limited.	Dose adjustment: Insufficient evidence/data for definitive dialysis-specific dosing. CV outcome evidence: Insufficient evidence/data. Safety: Limited dialysis-specific data.
Inclisiran	Dose adjustment: Not required. CV outcome evidence: LDL-C reduction established; cardiovascular outcome data pending. Safety: Injection-site reactions.	Dose adjustment: Not required in studied renal impairment. CV outcome evidence: LDL-C lowering preserved in CKD; cardiovascular outcome data pending. Safety: Generally well tolerated; injection-site reactions.	Dose adjustment: Insufficient evidence/data. CV outcome evidence: Insufficient evidence/data. Safety: Insufficient evidence/data.
Fibrates	Dose adjustment: Drug/formulation-specific; generally no adjustment at eGFR ≥ 60. CV outcome evidence: Limited and largely subgroup-restricted; overall benefit not consistent in broad populations. Safety: Myopathy, particularly with statins; serum creatinine may increase.	Dose adjustment: Drug-specific; dose reduction is required for some fibrates with declining eGFR, and use is generally avoided in severe CKD. CV outcome evidence: Limited and subgroup-restricted. Safety: Increased risk of adverse effects and serum creatinine elevation; caution with statin combinations.	Dose adjustment: Generally avoided. CV outcome evidence: Not established. Safety: Increased risk of adverse effects.
Icosapent ethyl	Dose adjustment: No renal adjustment specified. CV outcome evidence: Established in eligible statin-treated patients in REDUCE-IT. Safety: Atrial fibrillation/flutter and increased bleeding risk.	Dose adjustment: No renal adjustment specified. CV outcome evidence: Cardiovascular benefit preserved in studied CKD subgroups; evidence in advanced CKD is limited. Safety: Atrial fibrillation/flutter and bleeding.	Dose adjustment: Insufficient evidence/data. CV outcome evidence: Insufficient evidence/data. Safety: Insufficient evidence/data.
ApoC-III inhibitors	Dose adjustment: Drug-specific; renal dosing data are limited. CV outcome evidence: TG reduction demonstrated; cardiovascular outcome benefit not established. Safety: Agent-specific; safety profiles differ by pharmacological platform.	Dose adjustment: Insufficient evidence/data in advanced CKD. CV outcome evidence: Not established. Safety: Advanced CKD data are limited; agent-specific monitoring may be required.	Dose adjustment: Insufficient evidence/data. CV outcome evidence: Insufficient evidence/data. Safety: Insufficient evidence/data.
ANGPTL3 inhibitors	Dose adjustment: No renal adjustment specified for evinacumab. CV outcome evidence: LDL-C reduction established in HoFH; cardiovascular outcome benefit not established. Safety: Infusion reactions and hypersensitivity.	Dose adjustment: No adjustment specified in mild/moderate renal impairment; severe impairment data are limited. CV outcome evidence: Cardiovascular outcome benefit not established. Safety: Infusion reactions and hypersensitivity.	Dose adjustment: Insufficient evidence/data. CV outcome evidence: Insufficient evidence/data. Safety: Insufficient evidence/data.
Pelacarsen	Dose adjustment: Not established; investigational. CV outcome evidence: Lp(a) reduction demonstrated; cardiovascular outcome data pending. Safety: Injection-site reactions; long-term safety under investigation.	Dose adjustment: Not established. CV outcome evidence: No established CKD-specific cardiovascular outcome benefit; CKD subgroup data are limited. Safety: Limited CKD-specific data.	Dose adjustment: Not established. CV outcome evidence: Insufficient evidence/data. Safety: Insufficient evidence/data.
Olpasiran	Dose adjustment: Not established; investigational. CV outcome evidence: Lp(a) reduction demonstrated; cardiovascular outcome data pending. Safety: Injection-site reactions; long-term safety under investigation.	Dose adjustment: Not established. CV outcome evidence: No established CKD-specific cardiovascular outcome benefit. Safety: Limited CKD-specific data.	Dose adjustment: Not established. CV outcome evidence: Insufficient evidence/data. Safety: Insufficient evidence/data.
Lepodisiran	Dose adjustment: Not established; investigational. CV outcome evidence: Lp(a) reduction demonstrated; cardiovascular outcome data pending. Safety: Injection-site reactions; long-term safety under investigation.	Dose adjustment: Not established. CV outcome evidence: No established CKD-specific cardiovascular outcome benefit. Safety: Limited CKD-specific data.	Dose adjustment: Not established. CV outcome evidence: Insufficient evidence/data. Safety: Insufficient evidence/data.
Zerlasiran	Dose adjustment: Not established; investigational. CV outcome evidence: Lp(a) reduction demonstrated; cardiovascular outcome data pending. Safety: Injection-site reactions; long-term safety under investigation.	Dose adjustment: Not established. CV outcome evidence: No established CKD-specific cardiovascular outcome benefit. Safety: Limited CKD-specific data.	Dose adjustment: Not established. CV outcome evidence: Insufficient evidence/data. Safety: Insufficient evidence/data.
Muvalaplin	Dose adjustment: Not established; investigational oral agent. CV outcome evidence: Lp(a) reduction demonstrated; cardiovascular outcome data pending. Safety: Generally well tolerated in phase 2; long-term safety under investigation.	Dose adjustment: Not established. CV outcome evidence: No established CKD-specific cardiovascular outcome benefit. Safety: Limited CKD-specific data.	Dose adjustment: Not established. CV outcome evidence: Insufficient evidence/data. Safety: Insufficient evidence/data.

## Data Availability

No new data were created or analyzed in this study. Data sharing is not applicable to this article.
